# TSPY potentiates cell proliferation and tumorigenesis by promoting cell cycle progression in HeLa and NIH3T3 cells

**DOI:** 10.1186/1471-2407-6-154

**Published:** 2006-06-09

**Authors:** Shane W Oram, Xing Xing Liu, Tin-Lap Lee, Wai-Yee Chan, Yun-Fai Chris Lau

**Affiliations:** 1 Department of Medicine, VA Medical Center, University of California, San Francisco, USA; 2 Laboratory of Clinical Genomics, National Institute of Child Health and Human Development, National Institutes of Health, Bethesda, Maryland, USA; 3 Laboratory of Cell and Developmental Genetics, Department of Medicine, VA Medical Center, 111C5, 4150 Clement St, San Francisco, CA 94121, USA

## Abstract

**Background:**

TSPY is a repeated gene mapped to the critical region harboring the gonadoblastoma locus on the Y chromosome (GBY), the only oncogenic locus on this male-specific chromosome. Elevated levels of TSPY have been observed in gonadoblastoma specimens and a variety of other tumor tissues, including testicular germ cell tumors, prostate cancer, melanoma, and liver cancer. TSPY contains a SET/NAP domain that is present in a family of cyclin B and/or histone binding proteins represented by the oncoprotein SET and the nucleosome assembly protein 1 (NAP1), involved in cell cycle regulation and replication.

**Methods:**

To determine a possible cellular function for TSPY, we manipulated the TSPY expression in HeLa and NIH3T3 cells using the Tet-off system. Cell proliferation, colony formation assays and tumor growth in nude mice were utilized to determine the TSPY effects on cell growth and tumorigenesis. Cell cycle analysis and cell synchronization techniques were used to determine cell cycle profiles. Microarray and RT-PCR were used to investigate gene expression in TSPY expressing cells.

**Results:**

Our findings suggest that TSPY expression increases cell proliferation *in vitro *and tumorigenesis *in vivo*. Ectopic expression of TSPY results in a smaller population of the host cells in the G_2_/M phase of the cell cycle. Using cell synchronization techniques, we show that TSPY is capable of mediating a rapid transition of the cells through the G_2_/M phase. Microarray analysis demonstrates that numerous genes involved in the cell cycle and apoptosis are affected by TSPY expression in the HeLa cells.

**Conclusion:**

These data, taken together, have provided important insights on the probable functions of TSPY in cell cycle progression, cell proliferation, and tumorigenesis.

## Background

The testis specific protein Y-encoded (TSPY) gene was one of the early genes to be identified from the human Y chromosome [1,2]. TSPY is embedded in a 20.4-kb DNA fragment that is tandemly repeated ~35 times in humans [3]. The 2.8-kb TSPY transcriptional unit consists of six exons and 5 introns distributed primarily on the short arm of the Y chromosome [2,4]. The bovine Y chromosome contains 50–200 copies of TSPY, while the rat Y chromosome contains a single copy. The mouse possesses a nonfunctional Tspy gene, on its Y chromosome, that harbors several stop codons within its open reading frame [5-7]. The human TSPY is expressed in both fetal and adult testes [2,4,8]. It is localized in the cytoplasm and nucleus of embryonic gonocytes and adult spermatogonial cells [4,8]. In particular, the spermatogonial cells are the only cells in the male capable of entering both mitotic and meiotic cell division. The exact function of the TSPY gene product is thus far unknown. It has been hypothesized to regulate the normal proliferation of spermatogonia and marks the entry of the spermatogonia into the meiotic differentiation [9].

TSPY is expressed in adult testis as a phosphoprotein with an apparent molecular weight of 38 kD [4]. It harbors a SET/NAP domain, conserved among members of a protein family, represented by the SET oncoprotein and nucleosome assembly protein-1 (NAP-1) respectively. Major members of this protein family include SET, NAP-1, TSPY, differentially expressed nucleolar TGF-β1 target (DENTT) [10,11]/cell division autoantigen-1 (CDA1) [12]/TSPX [13]. SET was initially identified in a patient with acute undifferentiated leukemia, who harbored an intrachromosomal translocation on chromosome 9 [14-16] and demonstrated to bind B-type cyclins [17]. SET regulates the G_2_/M transition by modulating cyclin B-cyclin-dependent kinase 1 (CDK1) activity [18]. NAP-1 interacts with B-type cyclins in budding yeast and frogs [17]. In *Saccharomyces cerevisiae*, cells that lack NAP-1, the Clb2 (B-type cyclin) was unable to efficiently induce mitotic events [19,20]. Over-expression of SET or CDA1 results in an inhibitory effect on cell cycle progression at the G_2_/M phase [18], suggesting that SET/NAP-containing proteins are cell cycle regulators.

Deletion mapping for the gonadoblastoma locus on the Y chromosome (GBY) [21] has localized this oncogenic locus in a critical region (~1–2 Mb) on the short arm of this chromosome that contains most of the functional copies of the TSPY gene [22,23]. Elevated levels of TSPY protein have been observed in gonadoblastoma, thereby providing supporting evidence for TSPY as a likely candidate for the GBY [4,9,24,25]. TSPY is also expressed in testicular carcinoma-in-situ (CIS) [4,25], seminomas [24], prostate cancer specimens/cell lines [26-28], melanomas [29] and hepatocellular carcinoma [30]. To test the hypothesis that TSPY is involved in cell cycle regulation and its aberrant expression could contribute to the overall tumorigenesis, we have examined the effects of ectopic expression of TSPY in cell proliferation and tumorigenesis in athymic nude mice, using the tetracycline (Tet-off) regulation system in human HeLa and mouse NIH3T3 cells [31]. Our results suggest that ectopic expression of TSPY increases cell proliferation *in vitro *and tumorigenesis *in vivo*. Expression of TSPY expedites the transition of the cells through the G_2_/M phase of the cell cycle, indirectly up-regulates pro-growth genes and down-regulates apoptosis inducing molecules and growth inhibitory genes, thereby promoting cell proliferation in both cell cultures and whole animals.

## Methods

### Plasmids and stable cell transfection

The TSPY cDNA [2] was inserted at the EcoR1 site of the bicistronic vector, pTRE-IRES-GFP (designated as pTIG). The resulting construct (pTIG-TSPY) is capable of expressing both TSPY and EGFP under control of a modular tetracycline-responsive promoter. HeLa and NIH3T3 Tet-off cells harboring a stably integrated tetracycline transactivator gene were purchased from Clontech-BD BioSciences (Mountain View, CA). They were cultured in DMEM media containing 10% fetal bovine serum (FBS), 1% penicillin/streptomycin (P/S), and 400 μg/ml G418 (Invitrogen-Life Technologies, Carlsbad, CA) at 37°C in 5% CO_2_.

To generate stable cell lines conditionally expressing TSPY, the Tet-off cells were co-transfected with the pTIG-TSPY or pTIG vector and the hygromycin resistance marker, pTK-Hyg, at a ratio of 20:1, using either the Lipofectamine Plus (Invitrogen-Life Technologies, Carlsbad, CA) or FuGene6 (Roche, Alameda, CA) reagents, according to the instructions of the respective manufacturers. The cells were selected with the complete medium plus 300 μg/ml hygromycin (Invitrogen-Life Technologies, Carlsbad, CA) at a density of 4 × 10^5 ^cells per 100 mm culture dish. Cell colonies were selected with or without 2 ng/ml doxycycline (Dox) (Sigma, St. Louis, MO) in the selective media for 2–3 weeks. Positive colonies were isolated individually and clonally expanded as sub-lines. Alternatively, the colonies were pooled and isolated by preparative flow cytometry based on their EGFP expression using a FACSVantage SE Cell Sorter (Becton Dickinson, Frankilin Lakes, NJ) at the Laboratory for Cell Analysis, Cancer Center, University of California, San Francisco. Cells expressing EGFP were collected on ice in fresh media and immediately re-plated in culture dishes with complete media. Cells isolated with either strategy were then analyzed for their responsiveness to doxycycline regulation of their TSPY and EGFP transgenes using western blotting and fluorescence microscopy respectively.

### Immunofluorescence and western blotting

EGFP expression in cultured cells was observed directly under a Zeiss Axiophot fluorescence microscope using an excitation filter HQ 480/40 and an emission filter HQ 510 LP (Chroma Technology Corp., Rockingham, VT). TSPY expression was detected by indirect immunofluorescence according to established procedures. The cells were stained with a polyclonal TSPY antiserum [9] at 1:100 dilution at 4°C overnight, rinsed 3 times with PBS, incubated with a goat anti-rabbit IgG antibody conjugated to Texas Red (1:1000 dilution) for 30 minutes at room temperature, and analyzed with the Zeiss fluorescence microscope and appropriate filter set for Texas Red.

Western blotting of total cell lysates from HeLa or NIH3T3 Tet-off cells grown in the presence or absence of 2 ng/ml Dox was processed according to established procedures, using TSPY antisera and monoclonal antibodies, as described previously[32]. Immunoblot signals were detected by enhanced chemiluminescence (ECL) technique (Amersham, Piscataway, NJ).

### Cell proliferation analysis

Cell proliferation was analyzed by XTT assay, based on the cleavage of the tetrazolium salt XTT in the presence of an electron-coupling reagent by the succinate-tetrazolium reductase whose activity was directly associated with number of viable cells. HeLa or NIH3T3 Tet-off cells stably transfected with pTIG-TSPY or pTIG were grown on 100 mm dishes in culture media in the presence or absence of 2 ng/ml doxycycline for 3 days. Cells were then seeded at a concentration of 5 × 10^3 ^cells/well in 100 μl culture media with or without Dox on a 96-well microtiter plate, incubated at 37°C with 5% CO_2 _and analyzed in triplicates at 24 h intervals for 4 days. Fifty μl XTT labeling mixture was added to each well 4 hours before each spectrometric measurement.

### Colony formation assay

HeLa and NIH3T3 Tet-off cells were transfected with either pTIG-TSPY or pTIG plasmid and cultured for 3 weeks in a selective medium containing 300 μg/ml hygromycin and with or without 2 ng/ml Dox. The resulting colonies were stained with Giemsa and counted manually. Each 100 mm dish represented 1:10 dilution of cells from a single well, which had been transfected with 1 μg of TIG-TSPY or vector and 0.05 μg pTK-Hyg selectable marker.

### Tumorigenicity assay in athymic nude mice

Female 8-week old athymic nu/nu mice (Charles River Laboratories, Wilmington, MA) were used for tumorigenicity assays. Subconfluent and exponentially growing monolayer cells of each cell line were trypsinized, washed and resuspended in PBS. One million TSPY-transfected HeLa cells or 10 millions of similarly transfected NIH3T3 cells in 100 μl PBS were inoculated subcutaneously into the flanks of the nude mice. Control cells, transfected with pTIG or non-transfected parental cells, were inoculated similarly. Six animals were used for inoculation of each cell type. The mice were fed with drinking water with or without 2 μg/ml of doxycycline. Tumor growth was measured by the tumor volume, in mm^3^, estimated from the length (L) and width (W) of the tumors and the formula (L × W^2^)/2 [33]. The tumorigenicity assays were terminated by sacrificing the mice at 5 weeks (for HeLa cells) or 7 weeks (for NIT3T3 cells) after inoculation. All animal studies were conducted under an approved protocol by the Institutional Animal Care and Use Committee of the VA Medical Center, San Francisco.

### Cell synchronization and cell cycle analysis

Equal number of HeLa Tet-off cells stably transfected with either pTIG-TSPY or pTIG were grown overnight, washed with PBS, and fed with fresh media containing 2.5 mM thymidine (for G_1_/S synchronization) or 80 ng/ml colcemid (for M phase synchronization) [34,35]. Cells were synchronized in the respective media for 24 hours, washed 3 times with PBS and released into fresh complete media. At specific time points, the media was removed and the cells were washed in PBS, trypsinized, collected, washed again in PBS, placed in 1–3 ml of ice cold 70% ethanol and incubated at -20°C for 1 hour – overnight. Cells were then incubated in 10 μg/ml propidium iodide/0.1% Triton X-100/0.1% RNase in PBS solution at 37°C for 30 minutes in the dark. They were resuspended in 0.5 ml PBS and analyzed with a FACSCalibur flow cytometer at the Laboratory for Cell Analysis, UCSF Cancer Center. Cell cycle analysis was performed using ModFit LT (Verity Software House, Topsham, ME), FlowJo (Tree Star, Ashland, OR), and Openlab (Improvision, Lexington, MA) software.

### Microarray analysis of gene expression profiles

Total RNA was isolated from respective cell populations with TRIzol reagent (Invitrogen, Carlsbad, CA) and purified with RNeasy Mini Kit (Qiagen, Valencia, CA) in accordance with the manufacturer's instructions. The quality of RNA was assessed using the Agilent 2100 Bioanalyzer (Agilent Technologies, Palo Alto, CA). Five μg of RNA was converted into double-stranded cDNA using a cDNA synthesis kit (Affymetrix, Santa Clara, CA) with a special oligo(dT)_24 _primer containing a T7 RNA promoter site added 3' to the poly(T) tract. Biotinylated cRNAs were generated from cDNAs using the bioarray high yield RNA transcript labeling kit (Enzo Life Science, Farmingdale, NY) and subsequently purified with the RNeasy kit (Qiagen, Valencia, CA). Complementary RNA probe derived from each sample was fragmented and hybridized to GeneChip^® ^Human Genome U133 Plus 2.0 Array containing 47,000 transcripts and variants using the Affymetrix GeneChip Fluidics Station 450. Arrays were scanned by an Affymetrix Gene Scanner 3000, the image files were processed using Microarray Analysis Suite version 5.0 (Affymetrix, Santa Clara, CA). Each biological sample was analyzed with three technical replicates. All the 54,675 gene spots were filtered based on spot quality, statistical test and corrections before subsequent analyses. To identify differentially expressed transcripts, all spots were first normalized by scaling total chip fluorescence intensity to a common value of 100 prior to comparison, and a normalization value was set at 1. To minimize the potential false-positives using t-test alone, the t-test p-value of each gene when performing a statistical test was corrected by multiple testing corrections, which adjust the individual p-value for each gene to keep the overall error rate (or false positive rate) to less than or equal to a cutoff at p < 0.005. Multiple testing corrections based on Bonferroni step-down method were used. The corrected p-value is calculated and considered to be significant if it is less than 0.05. The false discovery rate applied in the tests was 5%. Data and statistical analyses were performed with Genespring 7.0 software (Silicon Genetics, Redwood City, CA).

Semi-quantitative RT-PCR was used to validate genes differentially expressed from data obtained with microarray analysis, as described previously [27]. The primers were derived from the corresponding cDNA sequences capable of amplifying roughly 100 to120-bp fragments from transcripts of the respective genes (Table 1). GADPH was included as a control in all the RT-PCR experiments. The number of cycle for each sample was independently determined to insure the reaction was terminated during the exponential phase of the amplification. PCR reaction products were separated on 1.5% agarose gels using ethidium bromide for visualization. The relative abundance of each PCR product was determined by quantitative analysis of digital photographs of the gels viewed under UV light (LabWorks software; UVP, Inc.).

## Results

### Tet-off regulation of TSPY gene expression

**Figure 1:**
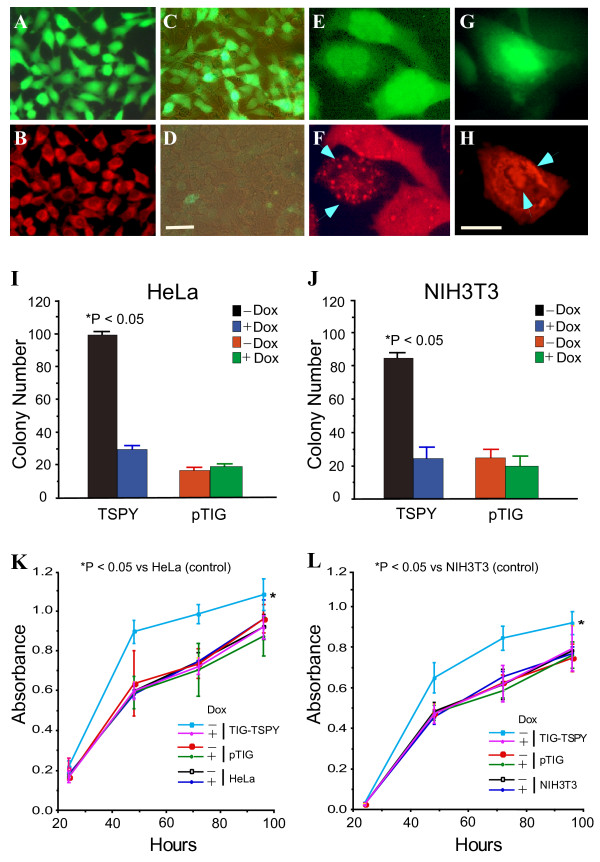
A-H) Tet-off regulation of TSPY and EGFP expression in HeLa cells. A bicistronic transgene capable of expressing both TSPY and EGFP in the same transcriptional unit was stably transfected to HeLa Tet-off cells. EGFP fluorescence (A) and TSPY (B) were detected by direct observation and immunofluorescence respectively. Such expression (i.e. EGFP in C) could be suppressed by administration of doxycycline in the culture media (D). EGFP was located in both cytoplasm and nuclei of the host cells (E, G) while TSPY could display scattered locations along the periphery and within the nuclei, in addition to its cytoplasmic distribution (F, H, blue arrows). I-L) Effects of TSPY expression in cell transfection efficiency and proliferation in HeLa and NIH3T3 Tet-off cells. HeLa (I) and NIH3T3 (J) Tet-off cells were co-transfected with either TIG-TSPY (TSPY) construct or the vector pTIG alone with the TK-Hyg plasmid and were selected in culture media with hygromycin with (+) or without (-) doxycyclinc (Dox). Both HeLa (I) and NIH3T3 (J) cells expressing TSPY (i.e. TSPY without doxycycline) consistently formed higher numbers of colonies than those repressing the same transgene (i.e. TSPY with doxycycline) while those expressing EGFP in the vector (pTIG) alone did not show any differences in the transfection efficiency in the absence or presence of doxycycline. Cell proliferation assays demonstrated that HeLa (K) and NIH3T3 (L) Tet-off cells over-expressing TSPY (without doxycycline) proliferated at a faster rate(s) than those repressing the TSPY gene (with doxycycline). Again, cells harboring the vector (pTIG) alone did not show any difference in their proliferative activities in media with or without doxycycline. Bars in D and H represent 40 μM in A-D and 10 μM in E-H respectively. * Student's t-test analysis indicated that there were statistical significance in differences between TSPY-expressing cells and vector-alone cells.

TSPY has been hypothesized to play a role(s) in regulation of germ cell proliferation and meiotic division. When it is ectopically expressed in cells incompatible with germ cell proliferation and/or division, it exerts a yet-to-be defined oncogenic effect(s) and contributes to the overall tumorigenic process(es) in susceptible cells of the affected organ/tissue [9,24,28]. To evaluate the effects of such ectopic TSPY expression, we had used the Tet-off transgene regulation system to manipulate its expression in HeLa and NIH3T3 cells [31]. HeLa cells are human female cervical carcinoma cells without the Y chromosome (and hence TSPY genes) while the NIH3T3 cells are normal (non-tumorigenic) mouse fibroblastic cells. Using the bicistronic vector, TIG, we were able to demonstrate that both TSPY and EGFP could be co-expressed in the same stably transfected cells (Figure 1A, B) and could be manipulated with the addition of doxycycline in the culture media (Figure 1C, D). EGFP was distributed relatively evenly among different compartments of most cells, while TSPY was located primarily in the cytoplasm and lightly in the nuclei. A small portion of cells, however, showed substantial nuclear localization or aggregated distribution of the TSPY protein (Figure 1F, H, arrows). Similar nuclear location of TSPY had been observed in various tumor specimens [9,25]. A recent report suggests that a CD2-dependent phosphorylation of the tyrosine reside at position 300 of the predominant TSPY isoform is essential for its nuclear translocation [36]. Currently, the significance of such differential distribution of TSPY in the cell compartments is uncertain. The present results demonstrate that the TSPY and EGFP are expressed abundantly and manipulated effectively with the Tet-off system in stably transfected HeLa and NIH3T3 cells.

### Over-expression of TSPY increases the colony formation efficiency and cell proliferation

To determine the effects of ectopic TSPY expression in cell growth, the transfection efficiency of the pTK-Hyg marker was determined in combination with either the TIG-TSPY construct or TIG vector in HeLa and NIH3T3 Tet-off cells. Approximately 3-fold higher numbers of colonies were observed in TIG-TSPY transfected cells selected without the doxycycline, i.e. over-expressing TSPY and EGFP, over identically transfected cells selected in media containing doxycycline, i.e. repressing the TSPY and EGFP expression (Figure 1I, J, left panel). Such differential efficiency of colony formation was absent in cells transfected with the TIG vector alone either with or without doxycycline in the selection media (Figure 1I, J, right panel). Although we cannot rule out completely that co-expression of TSPY might enhance the TK-Hyg gene expression, these results, and those from cell proliferation analysis described below, suggested that over-expression of TSPY enhances the efficiency of cell growth under such selection.

To evaluate the effects of TSPY expression in cell proliferation, TIG-TSPY, TIG transfected cells and the respective parental cells were analyzed with the XTT cell proliferation assay. Both HeLa and NIH3T3 Tet-off cells over-expressing TSPY-EGFP showed consistently 30–45% higher proliferative activities than those whose transgenes were repressed by doxycycline. Cells transfected with the vector alone, similar to the parental cells, did not show any proliferative differences in the presence or absence of doxycycline in the media (Figure 1K and 1L). These findings suggest that ectopic expression of TSPY potentiates cell proliferation in cultured cells.

### TSPY expression accelerates tumor growth in nude mice

**Figure 2:**
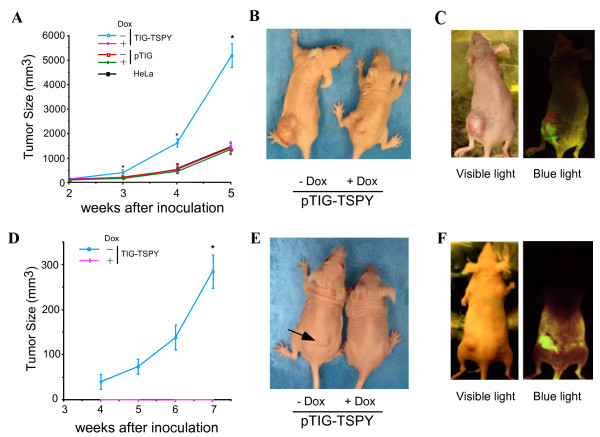
TSPY enhances tumor growth in athymic nude mice. Tumorigenicity assays demonstrated that HeLa Tet-off cells (A) over-expressing TSPY (- Dox) formed tumor at a faster rate in athymic nude mice than those whose TSPY expression was repressed with doxycycline (+ Dox) in the drinking water. Cells harboring the vector alone (pTIG) with or without doxycycline administration or the parental cells (HeLa) showed similar tumor growth rates as that of cells repressing TSPY transgene (+Dox) in the host animals. NIH3T3 Tet-off cells are non-tumorigenic cells that normally do not form tumor in athymic hosts. Inoculation of these cells over-expressing TSPY (D, – Dox), however, produced small size tumors in 5 of 6 nude mice (E) while no tumor was observed in animals inoculated with the same cells and fed with doxycycline-containing water (+Dox). Inoculation of NIH3T3 Tet-off cells harboring the vector alone or parental cells did not produce any tumor in the same hosts fed with drinking water with or without doxycycline (data not shown). B and E show examples of athymic nude mice from respective inoculations at the end of the experiments. Co-expression of EGFP provided a convenient means to observe directly the tumor growth (size) in nude mice (C, F). * Student's t-test analysis indicated that there were statistical significance in differences between TSPY-expressing cells and vector-alone cells.

Tumorigenicity assay was used to correlate the proliferative effects of TSPY in cultured cells to those in whole animals. HeLa Tet-off cells transfected with either TIG-TSPY or TIG vector alone and parental cells were inoculated subcutaneously on the flanks of female athymic nude mice. The mice were fed with drinking water either with or without doxycycline for 5 weeks. The size of tumors formed at the inoculation site was measured with standard procedure at the respective time points (Figure 2A, B). An accelerated tumor growth was observed in mice inoculated with HeLa Tet-off cells over-expressing TSPY (without doxycycline in their drinking water) compared to the group repressing TSPY (with doxycycline in their drinking water). No accelerated tumor growth was observed in the parental or the groups inoculated with HeLa cells harboring the vector alone, with or without doxycycline in their drinking water. These results suggested that ectopic expression of TSPY increased tumor growth in athymic nude mice.

NIH3T3 cells are established mouse fibroblasts that normally do not form tumors in athymic animals. Using a similar tumorigenicity assay, small size tumors were observed in 5 out of 6 mice inoculated each with about 10 millions cells harboring and expressing TIG-TSPY (without doxycycline in drinking water), while no tumors were observed in the group of animals inoculated with similar number of transfected cells, but fed with doxycycline in their drinking water (Figure 2D, E). Again, mice inoculated with the NIH3T3 cells transfected with vector alone or the parental cells did not show any tumor formation, with or without doxycycline in their drinking water. The expression of the TSPY-EGFP transgene in the tumors of these nude mice, fed with normal drinking water, could be confirmed by direct observation of EGFP fluorescence in the whole animals (Figure 2C, F).

### TSPY expedites a rapid transition of G_2_/M of the cell cycle

**Figure 3:**
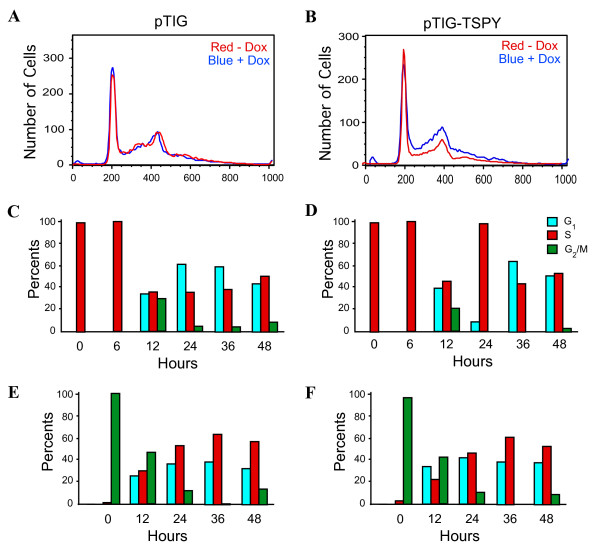
TSPY accelerates G_2_/M transition in cell cycle. Flow cytometry analysis of HeLa Tet-off cells harboring either the vector, pTIG, (A) or pTIG-TSPY (B) construct showed a reduced number of cells at G_2_/M phase in cells over-expressing TSPY (B, -Dox) than those repressing TSPY (B, +Dox) or harboring the vector alone (A, +/- Dox). To evaluate the probable reason(s) for such differential distribution of cells in various phases of the cell cycle, HeLa Tet-off cells harboring either the vector alone (C) or pTIG-TSPY construct (D) were synchronized at G_1_/S, released into S phase and analyzed similarly with flow cytometry at 0, 6, 12, 24, 36 and 48 hours after the release. HeLa cells harboring pTIG-TSPY construct (D) progressed at a faster rate than those harboring the vector alone (C), and reached in the second S phase within 24 hours after release from the synchronization while significant number of cells harboring the vector alone (C) remained in G_1 _phase at the same time. Such synchronized cell cycle progression disappeared towards 36 and 48 hours in both cell populations (C, D). When the cells were synchronized at the G_2_/M phase, released into mitosis, and analyzed similarly with flow cytometry, such accelerated rate of cell cycle progression were not observed readily between populations harboring the vector alone (E) and TSPY (F). Again, cells over-expressing TSPY showed a reduced number(s) at G_2_/M (e.g. 24 and 36 hour time points in D and all time points in F as compared to those in C and E respectively) when they were over-expressing TSPY, resembling those of asynchronous cells (B).

Members of the SET/NAP family of proteins had been demonstrated to bind type B cyclins and exert biological effects on cell cycle progression. Our study, so far, showed that ectopic expression of TSPY in HeLa and NIH3T3 cells potentiated cell proliferation in vitro and tumor growth in nude mice. To evaluate the likely cell cycle stage(s) in which TSPY affected its pro-growth function, both exponentially growing and synchronized HeLa Tet-off cells harboring either the TIG-TSPY transgene or TIG vector alone were analyzed with flow cytometry techniques. Exponentially growing HeLa Tet-off cells harboring the vector alone showed similar patterns in their cell cycle distribution when they were grown in the presence or absence of doxycycline (Figure 3A) while those over-expressing TSPY showed a small portion of its cells in the G_2_/M phase (Figure 3B, red line). When expression of the TSPY transgene was repressed with doxycycline in the media (Figure 3B, blue line), the distribution of cells among the different stages of the cell cycle was similar to those of control cells (Figure 3A).

To determine why there was fewer number of TSPY expressing HeLa Tet-off cells at G_2_/M phase, as compared to HeLa Tet-off harboring the vector alone, the cells were examined further with cell synchronization and cell cycle analysis with flow cytometry. Cells were synchronized at the G_1_/S boundary, released into the S phase and analyzed with flow cytometry thereafter at 0, 6, 12, 24, 36 and 48 hours. The relative percents of the cells distributed at different stages (G1, S and G_2_/M) of the cell cycle were determined from the respective flow cytometry charts. At 6 hours, most cells in both populations were mostly at S phase while at 12 hours, some cells had gone through S and G_2_/M and reached G1. However, the majority of the cells over-expressing TSPY reached the second S phase again in 24 hours (Figure 3D) while cells harboring just the vector alone transited the cell cycle stages more slowly and had only ~35% of cells reaching the second S phase (Figure 3C). The synchronization effects seemed to dissipate at 36 and 48 hours in both populations (Figure 3C, D) and the distributions of their cells in various cell cycle stages resembled those of exponentially growing cells (as in Figure 3A, B respectively). Since cells over-expressing TSPY showed a smaller number in G_2_/M, such rapid transition of the cell cycle after the G_1_/S phase synchronization suggested that the cell could progress through the G_2_/M phase faster than those harboring the vector alone. To confirm our postulation that TSPY mediated a fast transit through G_2_/M, both HeLa cell populations were synchronized at metaphase by colcemid treatment, released into cell cycle and analyzed thereafter at 0, 12, 24, 36, and 48 hours with flow cytometry. Our results showed that cells expressing TSPY progressed through the G_1 _and S phases (Figure 3F) at similar rates to those lacking TSPY expression (Figure 3E). Again, at 36 and 48 hours after their release, cells in both populations (i.e. harboring TIG-TSPT and TIG) showed a similar cell cycle stage distribution, resembling those of exponentially growing and asynchronous cells. We surmise that the facilitating function of TSPY in the cell cycle was less obvious because the synchronization was at metaphase, immediate beyond the G_2 _stage in which TSPY is postulated to be effective in expediting cell cycle progression. Nevertheless, one could still observe fewer percents of cells were at G_2_/M in cells over-expressing than those lacking TSPY at these time points, thereby supporting a role of TSPY at this stage of the cell cycle, as discussed above.

**Figure 4:**
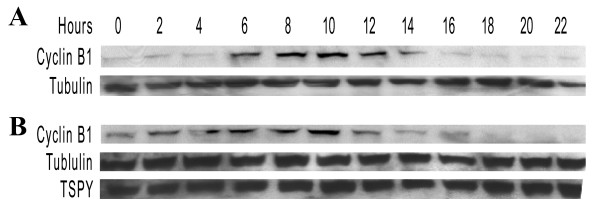
The rapid G_2_/M transition of HeLa cells over-expressing TSPY is associated with an early degradation of the mitotic cyclin B1. HeLa Tet-off cells harboring the vector alone (A) and TIG-TSPY construct (B) were synchronized at G_1_/S, released into S phase, harvested and analyzed with western blotting with various antibodies at 2-hour intervals for 22 hours. The mitotic cyclin B1 is synthesized normally in late S phase and G_2 _and is rapidly degraded before the cell exits mitosis. In cells over-expressing EGFP in the vector alone, the levels of cyclin B1 increased at 6 hours after the G_1_/S release, peaked at 10 hours and gradually decreased until 14–16 hours (A, top row) while in those over-expressing both EFGP and TSPY, the levels of cyclin B1 increased gradually at 2 hours after the G11/S release, peaked at 10 hours and rapidly reduced thereafter (B, top row). The filters were re-probed with tubulin antibody (A, bottom row; B, middle row) showing relatively even loading of the samples. TSPY was uniformly expressed and detected in samples of cells over-expressing this transgene (B, bottom row).

Cyclin B1 is a mitotic cyclin whose expression is primarily at late S and G_2 _[37,38]. Its degradation by the anaphase promoting complex is essential for the cell to exit mitosis [39-42]. Hence, the amount of cyclin B1 varies with the different stages of the cell cycle, i.e. the lowest at G_1 _and highest at G_2_/M. To substantiate the effects of TSPY in cell cycle progression, we had examined the relative amount of cyclin B1 in HeLa Tet-off cells harboring either TIG-TSPY or TIG vector alone, after they were synchronized at G_1_/S and released into the cell cycle. Cells were harvested at 2 hour-intervals, starting at 0 hours and ending at 22 hours. Total protein extracts were prepared from these cells and processed for western blotting with cyclin B1, tubulin and TSPY antibodies. Both cell populations showed minimal level of cyclin B1 at 0 hours immediately after release from G_1_/S phase synchronization. The levels of cyclin B1 in HeLa cells harboring the vector alone increased gradually starting at 6 hours and peaked at 10 hours and gradually declined through detectable levels to 14–16 hours (Figure 4A). Cells expressing TSPY showed a detectable level immediately after release from G_1_/S synchronization (Figure 4B). The level increased gradually and peaked at 10 hours, but fell rapidly, although still detectable, thereafter at 12–14 hours. Repeat probing of the same/similar filters with a tubulin antibody showed that the loading was relatively even among the various samples (Figure 4A, lower panel, 4B, middle panel). Cell harboring the TIG-TSPY transgene expressed TSPY at consistent level throughout the cell cycle (Figure 4B, lower panel). These findings suggested that TSPY expressing cells exited the G_2_/M phase faster than those lacking such expression.

### Differential expression of growth-related genes in HeLa cells over-expressing TSPY

**Figure 5:**
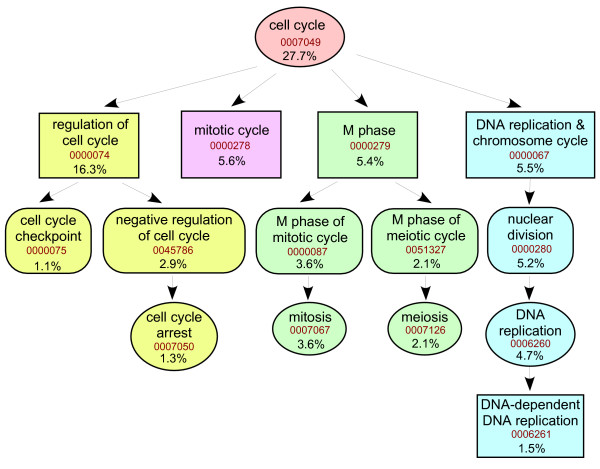
Diagrammatic illustration of various cell cycle-related biological processes in HeLa cells affected by TSPY over-expression, compared to those harboring the vector alone, as revealed by the GOTM program. Each process is represented by the respective GO accession number (red) and percents of genes within the 181 differentially expressed genes used in the GOTM analysis.

To determine how TSPY expression affects cell growth and cell cycle progression, microarray analysis was performed on HeLa Tet-off cells expressing TIG-TSPY and compared with those harboring the TIG vector alone, using the Affymetrix GeneChip containing 47,000 human transcripts and variants. Our results showed that TSPY and a limited number of genes were differentially expressed between the two cell populations. TSPY was expressed at the highest level while others were consistently affected at modest levels. The present findings were consistent with the fact that the two HeLa cell populations were almost identical, except the high TSPY expression in one and not the other. To translate sets of differentially regulated genes at each stage into functional profiles, 181 differentially expressed genes were analyzed with the web-based program, Gene Ontology Tree Machine (GOTM) [43]. Statistical analysis was performed to identify the most important Gene Ontology categories for the input gene sets and to suggest their potential biological importance in the categories. Three biological processes, cell cycle, phosphate transport, and neuromuscular development, were preferentially represented among the differentially expressed genes. Both cell cycle and phosphate transport could be related to cell growth, and hence are relevant differences between TSPY over-expressing and vector alone cell populations. The exact nature of differential expression of genes involved in neuromuscular development is uncertain. However, previous transgenic mouse studies demonstrated that the human TSPY promoter could be preferentially active in neurons of pre- and postnatal brains, suggesting a possible function of this Y chromosome gene in neural development [32]. The various cell cycle related processes affected by the constitutive expression of TSPY are illustrated (Figure 5). Table 2 lists 25 cell cycle related genes whose expression was either up- or down-regulated by over-expression of TSPY. Among the up-regulated genes were several oncogenes (epidermal growth factor receptor (ERBB) and members of the WNT (WNT5A) and RAS families (RAP1A)), growth factors (PDGFC, EGF-related, ANKRD15, RGC32, NANOS1), cyclin D2 (CCND2), a co-factor for the hypoxia inducible factor 1A (EP300), an apoptosis inhibitor (GSPT1) and an antigen (CD24) highly expressed in small cell lung carcinoma. The down-regulated genes included an inhibitor for CDK4/CDK6 (CDKN2B), transforming growth factor β3, pro-apoptotic factors (CLU and IGFB3), and an inhibitor of MAP kinases (DUSP5). In particular, the CCND2 gene (encoding cyclin D2) resides on chromosome 12p that is frequently amplified and expressed at high levels in testicular germ cell tumors. Cyclin D2 complexes with CDK4 or CDK6 to mediate G_1_/S transition and promote cell proliferation. Indeed CCND2 expression was up-regulated in HeLa Tet-off cells over-expressing TSPY. Conversely, an inhibitor, CDKN2B, against activation of CDK4/CDK6 was down-regulated in the same cells, thereby further supporting the possible role of TSPY in the CCND2-CDKN2B cell cycle regulation. In additional to CCND2, another genes, the transmembrane and tetratricopeptide repeats (TMTC1) from chromosome 12p was also up-regulated by the ectopic expression of TSPY in HeLa cells. Currently the function of TMTC1 is unknown. Other tetratricopeptide repeats-containing proteins, such as subunits cdc16, cdc23 and cdc27 of the anaphase promoting complex (APC), could play important roles in protein-protein interaction, cell division and receptor signaling [44-46].

**Figure 6:**
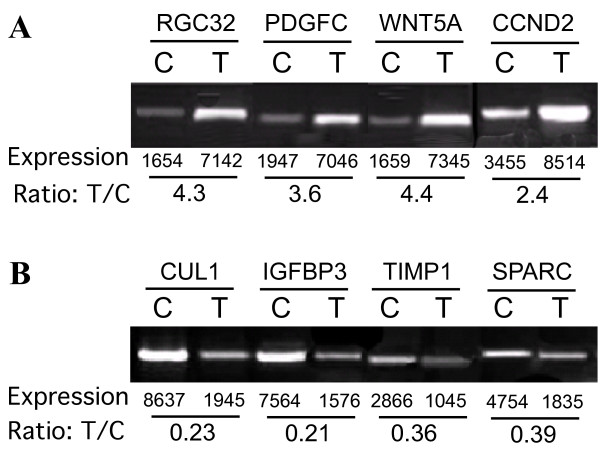
Semi-quantitation RT-PCR analysis of genes up-regulated (A) and down-regulated (B) in HeLa Tet-off cells over-expressing TSPY, using gene-specific primers (Table 1). C = cells stably transfected with vector (pTIG) alone; T = cells stably transfected with TIG-TSPY. The relative expression was calculated by digitization and quantitation software (LabWorks, UVP Inc.). The ratio of T/C represents the relative expression level between the TSPY expressing and control cells for the respective genes (see Table 2).

To confirm the microarray data, semi-quantitative RT-PCR was performed on 4 of the up-regulated genes and 4 of the down-regulated genes from Table 2 (bold typed). Figure 6A showed the results of the RT-PCR from HeLa Tet-off cells harboring either pTIG or pTIG-TSPY. RGC32, PDGFC, WNT5A, and CCND2 were confirmed as up-regulated genes in cells over-expressing TSPY; each showed a slight increase in fold expression compared to the microarray data. CUL1, IGFBP3, TIMP1, and SPARC were confirmed as down-regulated genes in cells expressing TSPY (Figure 6B); each of these also showed a slight change in fold expression compared to the microarray data. These results confirmed that cells expressing TSPY could indirectly up-regulate genes involved in the cell growth and proliferation and down-regulate genes involved in the cell cycle inhibitors, growth suppressors, and pro-apoptotic factors.

## Discussion

TSPY is a unique gene in the human genome. Although its transcriptional unit is only approximately 2.8-kb in size, it is embedded in a 20.4-kb tandemly repeated unit that shows >98% homology among the members of the TSPY gene family [47]. Numerous studies suggest that most of the repeat units are functional capable of coding for a variety of polymorphic TSPY proteins [28,48,49]. For one sequenced individual, the TSPY repeat units constitutes ~0.7 MB of DNA on the short arm and only one single copy on the long arm of the Y chromosome [48,50,51]. Currently, the exact nature of such tandem repetition of a functional gene and its flanking sequences is uncertain. However, we surmise that TSPY repeats could be hot spots for genetic rearrangements and/or transcriptional dysregulation, resulting in ectopic expression of TSPY variant transcripts and proteins in tissues that normally do not express this Y-located gene [9,29,30] and variation in copy numbers and/or genetic rearrangements [52].

TSPY is a member of a protein superfamily that is defined by a conserved 191 amino acid domain, designated as SET/NAP domain. In yeast, Nap1 can interact specifically with the B-type cyclin, Clb2, to mediate normal mitotic functions in fission yeast and to suppress polar bud growth in budding yeast [19,20]. The mammalian SET protein can interact with B-type cyclins and has been shown to regulate the G_2_/M transition by modulating cyclin B-cyclin-dependent kinase 1 (CDK1) activity [18]. Over-expression of either SET or CDA1 arrested cells at G_2_/M phase [12,18], an opposite effect to that of over-expression of TSPY, in HeLa cells, observed here. TSPY protein shares significant homology with these proteins at the SET/NAP domain, but lacks the C-terminal acidic tail that is found in NAP-1, SET and CDA1. Significantly, a recent study suggests that the X-located CDA1/DENTT is a homologue of TSPY and been re-designated as TSPX gene [13]. It shares significant similarities in exon organization, except additional exons at both its 5' and 3' termini. Deletion of the acidic domain in CDA1/TSPX eliminates its inhibitory effects on the G_2_/M progression in the cell cycle [12], suggesting the TSPY and TSPX might possess contrasting functions on cell cycle modulation.

The main physiological function of TSPY is currently uncertain. TSPY is expressed in embryonic germ cells and primarily in spermatogonia and to a reduced level the spermatids of adult testis [2,4,8,32]. Spermatogonia are a subset of cells in the testis that are capable of entering both mitotic and meiotic cell divisions [53]. Hence, TSPY has been postulated to serve a physiological function(s) in stem germ cell proliferation and in directing the spermatogonial cells to enter male meiosis [4,9]. Location of TSPY gene cluster in the critical region for GBY, the only oncogenic locus on the Y chromosome, establishes it to be a significant candidate for this special form of germ cell tumor. Indeed, TSPY has been detected in high levels in gonadoblastoma tissues as well as those of testicular germ cell tumors, more common forms of germ cell tumors [9,24,25]. Additional studies have demonstrated that TSPY is also expressed in prostate cancers and the androgen responsive LNCaP prostate cancer cell line [9,26], hepatoma specimens [30], and melanoma samples and melanoma cell lines [29]. The latter studies further substantiate the possible role of TSPY in human oncogenesis.

The present studies were designed to address the question on the effects of ectopic TSPY expression in cell proliferation and tumorigenesis in immunodeficient mice. Using the HeLa, a human (female) cervical carcinoma, and NIH3T3, a non-tumorigenic mouse (lacking a functional Tspy gene), cell lines and the Tet-off transgene regulation system, we demonstrated that over-expression of TSPY potentiates cell proliferation in culture and tumorigenicity in nude mice. Significantly, NIH3T3 cells are non-tumorigenic, the development of small tumors in nude mice inoculated with TSPY expressing NIH3T3 cells suggests that TSPY could potentially play the role of an oncogene. Our cell cycle analyses demonstrated that TSPY was capable of mediating the transition of its host cells through G_2_/M phase at a faster pace than those lacking TSPY. These findings, taken together, support the notion that TSPY is a growth-promoting gene that increases cell proliferation *in vitro *and tumorigenesis *in vivo*, thereby providing a possible explanation of abundant TSPY expression in tumor tissues.

Global transcriptional profiling with microarray analyses further supports the hypothesis that TSPY exerts pro-growth and proliferative functions in the cell cycle progression of its host cells. The up-regulation of such pro-growth genes and oncogenes and down-regulation of growth inhibitors and apoptotic factors in cells over-expressing TSPY, as revealed by the microarray studies, could be confirmed by semi-quantitative RT-PCR analysis. It is also possible that the differential gene expression is a result of indirect effects, perhaps through its interaction(s) with signaling molecules or cell cycle regulators.

## Conclusion

Although TSPY expression has been observed in gonadoblastoma, testicular germ cell tumors, prostate cancer, hepatomas, and melanomas, no studies have defined its probable role in human oncogenesis. The present studies have demonstrated that ectopic TSPY expression expedites cell cycle progression through shortening of the G_2_/M transition. TSPY also up-regulates pro-growth genes/oncogenes and down-regulates cell cycle inhibitors/apoptotic factors; however, whether this is a direct or indirect effect is unknown. Together, these findings provide a possible mechanism by which TSPY, in collaboration with other oncogenic events, contributes to tumorigenesis in dysfunctional germ cells and/or susceptible somatic cells/tissues.

## Abbreviations

TSPY, testis-specific protein Y-encoded; NAP, nucleosome assembly protein; DENTT, differentially expressed nucleolar TGF-β1 target; CDA, cell division autoantigen; CDK, cyclin-dependent kinase; GBY, gonadoblastoma locus on the Y chromosome; CIS, carcinoma in situ; DOX, doxycycline; DMEM, Dulbecco's minimal essential media.

## Competing interests

The author(s) declare that they have no competing interests.

## Authors' contributions

Y-FCL conceived the hypothesis that TSPY exerts a pro-growth function on cell cycle, supervised and coordinated this study and managed the overall research program on TSPY. SO conceived and performed the studies on growth and colony formation, cell cycle and cell synchronization, and cyclin B1 analysis with respect to cell cycle stages. XXL performed the XTT cell growth and nude mice tumorigenicity assays. TLL and WYC performed the microarray, bioinformatics and semi-quantitative RT-PCR analyses.

## Pre-publication history

The pre-publication history for this paper can be accessed here:



## Figures and Tables

**Table 1:** Gene-specific primers used in semi-quantitative RT-PCR analysis

Gene	Primers	Sequences
**RGC32**	Forward	GAGCGCCACTTCCACTACG
	Reverse	AGTGAATCTGCACTCTCCGAG
**PDGFC**	Forward	ACTCAGGCGGAATCCAACC
	Reverse	CTTGGGCTGTGAATACTTCCATT
**WNT5A**	Forward	TTGGTGGTCGCTAGGTATGAA
	Reverse	AGTGGCACAGTTTCTTCTGTC
**CCND2**	Forward	AGGCGGTGCTCCTCAATAG
	Reverse	TATCCCGCACGTCTGTAGGG
**CUL1**	Forward	ACCAGTCAAACCAAGCACGAG
	Reverse	GTCTGCCCCTTTTTCGACTTAG
**IGFBP3**	Forward	AGAGCACAGATACCCAGAACT
	Reverse	TGAGGAACTTCAGGTGATTCAGT
**TIMP1**	Forward	CTTCTGCAATTCCGACCTCGT
	Reverse	CCCTAAGGCTTGGAACCCTTT
**SPARC**	Forward	AGCACCCCATTGACGGGTA
	Reverse	GGTCACAGGTCTCGAAAAAGC

**Table 2:** List of genes identified in the microarray analysis between HeLa Tet-off cells harboring pTIG-TSPY and those harboring the vector pTIG alone

**Gene**	**GenBank ID**	**Expression**	**Remarks**
TSPY1	NM_003308	26.5	Testis specific protein, Y-linked, stably transfected gene in the cells
MAP2	NM_002374	3.41	Microtubule-associated protein 2, neurogenesis and tumorigenesis
**RGC32**	NM_014059	**2.56**	**Response gene to complement 32, deregulation of cell cycle required for tumor cell growth**
**PDGFC**	NM_016205	**2.32**	**Platelet derived growth factor C, a mitogenic factor**
MAP1B	NM_005909	1.93	Microtubule-associated protein 1B, structural protein, neural development, predictor for breast cancer
EFEMP1	NM_004105	1.79	EGF-containing fibulin-like extracellular matrix protein 1, growth factor
G1P2	NM_005101	1.72	α-interferon inducible protein, IFI-15K, facilitates viral growth
LUM	NM_002345	1.62	Lumican, epithelial cell migration and tissue repair
NANOS1	NM_199461	1.57	Human nanos homolog (of Drosophila), germ stem cell development expressed in spermatogonia and spermatocytes in human testis
**WNT5A**	NM_003392	1.55	**Wingless-type MMTV integration site family member 5A, Wnt gene family, implicated in oncogenesis and embryogenesis**
CD24	NM_013230	1.55	Small cell lung carcinoma cluster 4 antigen, high expression in SCLC
TMTC1	NM_175861	1.53	Transmembrane and tetratricopeptide repeats containing 1, multi-functional, possibly involved in regulation of cell cycle and/or mitosis.
**CCND2**	NM_001759	**1.46**	**Cyclin D2 complexes with CDK4 or CDK6, G1/S transition, amplified and over-expresses in numerous types of tumors**
RAP1A	NM_002884	1.44	RAS oncogene family member
EGFR	NM_005228	1.41	Epidermal growth factor receptor (ERBB oncogene), cell proliferation, amplified and expresses at high levels in many tumors
ANKRD15	NM_015158	1.38	Ankyrin repeat domain 15, tumor cell growth in renal cell carcinoma
GSPT1	NM_002094	1.32	G1 to S phase transition 1, an inhibitor of apoptosis
EP300	NM_001429	1.26	E1A binding protein p300, a co-factor for hypoxia inducible factor 1A
CDKN2B	NM_004936	0.71	Cyclin-dependent kinase inhibitor 2B (p15), binds to and prevents CDK4/CDK6 activation and G1/S progression, tumor suppressor
**CUL1**	NM_003592	**0.68**	**Cullin 1, involves in deneddylation and modulates G1/S transition**
TGFB3	NM_003239	0.67	Transforming growth factor, b3, suppresses tumor formation and blocks cell cycle progression
PTN	NM_002825	0.66	Pleiotrophin, neurite growth promoting factor 1, down-regulated in breast cancer
**TIMP1**	NM_003254	**0.64**	**Tissue inhibitor of metalloproteinase 1, suppresses tumor growth and metastasis**
DUSP5	NM_004419	0.58	Dual specificity phosphatase 5, inactivates kinases by dephosphorylation, and negatively regulates MAP kinases
**SPARC**	NM_003118	**0.56**	**Secreted protein, acidc cysteine-rich (osteonectin), loss of expression in lung cancers, regulates cell proliferation**
CLU	NM_001831	0.56	Clusterin, proapoptotic in colon cancer, down-regulated in CaP
**IGFBP3**	NM_000598	**0.49**	**Insulin-like growth factor binding protein 3, pro-apoptotic, its inactivation or repression is essential for growth of various tumors**

Genes listed in **boldface **were analyzed with semi-quantitative for RT-PCR (Figure 6).
